# The Impact of Physical Activity on the Development of Postpartum Depression

**DOI:** 10.1155/2024/6539734

**Published:** 2024-08-13

**Authors:** Marija Rovcanin, Ana Tomic, Sandra Sipetic Grujicic, Svetlana Jankovic, Bojana Ivic, Milan Lackovic, Maja Lackovic, Isidora Vujcic

**Affiliations:** ^1^Clinic for Gynecology and Obstetrics, Narodni Front, Kraljice Natalije 62, Belgrade 11000, Serbia; ^2^Center for Radiology and Magnetic Resonance Imaging, University Clinical Center of Serbia, Belgrade 11000, Serbia; ^3^Faculty of Medicine, University of Belgrade, Dr Subotica Starijeg 8, Belgrade 11000, Serbia; ^4^Institute of Epidemiology, Faculty of Medicine, University of Belgrade, Visegradska 26, Belgrade 11000, Serbia; ^5^University Hospital Center Dr Dragiša Mišović, Heroja Milana Tepica 1, Belgrade 11000, Serbia; ^6^Clinic for Psychiatry, University Clinical Centre of Serbia, Belgrade 11000, Serbia

## Abstract

Although the benefits of physical activity (PA) on mental well-being are well established, the vulnerability of women during pregnancy and the postpartum period poses challenges in studying the effects of PA on postpartum depression (PPD). This study investigated the association between total and domain-specific PA levels during pregnancy and postpartum depressive symptoms. The study included 105 healthy pregnant women whose PA status was evaluated by the Serbian version of the Physical Activity Questionnaire during Pregnancy (PPAQ-SRB) and their postpartum mental health by the Edinburgh Postnatal Depression Scale. Multivariable logistic regression was used to explore the independent relationship between PPAQ-SRB scores and the prediction of the PPD symptom occurrence. Our analysis revealed that the development of PPD symptomatology was associated with a lower “Total PPAQ-SRB score” (odds ratio (OR) 0.81; 95% confidence interval (CI) [0.70–0.93]; *p*=0.03) and “Total Activity score” (OR 0.82; 95% CI [0.71–0.93]; *p*=0.03) as well as lower scores of light-intensity PA (OR 0.81; 95% CI [0.69–0.96]; *p*=0.013), moderate-intensity PA (OR 0.82; 95% CI [0.71–0.94]; *p*=0.005), “Household/care” (OR 0.85; 95% CI [0.73–0.98]; *p*=0.028), and “Occupational” activities (OR 0.80; 95% CI [0.78–0.95]; *p*=0.012). Lower levels of light-to-moderate-intensity household and occupational activities appeared to increase the risk of PPD, indicating the importance of circumstances under which PA is performed. Hence, our findings indicate that PA during pregnancy can mitigate mood disorders in postpartum mothers. Moreover, reduced participation in light-to-moderate-intensity household and occupational activities seemed to increase the risk of PPD.

## 1. Introduction

Postpartum depression (PPD) is a heterogeneous illness, both in terms of manifested symptoms and the circumstances under which they emerge [1, 2].

The Diagnostic and Statistical Manual of Mental Disorders-Fourth Edition (DSM-IV) establishes a timeframe of 4 weeks following childbirth as the threshold for identifying the postpartum onset of PPD [3]. In contrast, the International Classification of Diseases-Tenth Revision (ICD-10) categorizes mental disorders as related to the puerperium if they commence within 6 weeks after delivery [4]. Moreover, a group of experts from different countries assembled in Sweden previously proposed that the “postnatal onset” be defined as a period of 3 months post-birth. This was influenced by the findings of Kendell et al. [5], showing that there is an elevated risk of hospitalization for various psychiatric disorders within the first 90 days following childbirth. Recently, the modification from the diagnostic label “postpartum depression” in DSM-IV to “peripartum onset” in DSM-Fifth Edition is based on empirical findings indicating that approximately half of the episodes of depression commonly referred to as PPD typically manifest before childbirth [6]. Accordingly, the American College of Obstetricians and Gynecologists defines perinatal depression as the experience of both severe and moderate depressive episodes during pregnancy or the first year after delivery [7].

Several epidemiological studies have been conducted worldwide on the prevalence, course, and nature of PPD. A recent study that included both a systematic review and a meta-analysis found that the overall prevalence of PPD was 14.0%. Furthermore, depending on the country's income classification, the prevalence ranges from 5% to 26.3% [8]. According to a previous study conducted in Serbia, 11.8% of postpartum women had signs of PPD [9].

Psychopathology during and after pregnancy can be attributed to multiple interconnected factors. Moreover, the coexistence of diverse risk factors is anticipated to enhance women's vulnerability to PPD. These include a positive family history of affective disorders and other psychiatric illnesses [10, 11, 12, 13] and PPD [14], a personal history of previous depressive episodes or other psychiatric illness [10], a personal history of PPD [11], and premenstrual dysphoric disorder [15]; stressful life events during pregnancy or in the early postpartum period [12, 16]; feeling of depression or anxiety [12, 17]; and perceived social isolation (or lack of social support) during pregnancy [18] and having a low level of social support during the postpartum [19, 20]. Additional potential risk factors associated with the onset of depressive symptoms include cesarean delivery [21], poor sleep patterns immediately before childbirth [22], heightened weight gain during the preceding pregnancy [23], multiparity [18], strained partner relationships [11], stress related to impending childcare responsibilities, challenges in emotional acceptance when faced with a newborn with an intense temperament, and unplanned or undesired pregnancy [24].

Owing to the vulnerability of women during pregnancy and the postpartum period, evidence-based interventions for PPD are primarily confined to pharmacotherapy, cognitive and interpersonal supportive therapy, psychosocial support through support groups, and complementary therapies [25]. The implementation of PA as an intervention is based on a limited number of studies that could offer a certain level of evidence for group exercise interventions and exercise counseling with the personal choice of exercise as an effective treatment for reducing PPD symptoms. Therefore, in light of PA intervention, the majority of randomized controlled trials (RCTs) reviewed in a recently published meta-analysis found improvements in postpartum depressive symptoms with 30 min of moderate-intensity activity performed three to five times per week for 4–6 months [26]. Moreover, another dose–response meta-analysis identified physical activity (PA) as a potentially advantageous intervention to decrease the likelihood of PPD, particularly since engaging in 90 min of PA per week could effectively reduce the risk of PPD [27].

Our objective was to investigate the association between total and domain-specific PA during pregnancy and PPD symptoms in healthy Serbian women who had a vaginal delivery.

## 2. Materials and Methods

### 2.1. Study Design

This study included 105 healthy women between the ages of 18 and 40 who received perinatal and obstetrical care at the Obstetrics and Gynecology Clinic, “Narodni Front,” in Belgrade, Serbia, from October 2020 to March 2021.

### 2.2. Participants

Healthy women who had experienced uncomplicated singleton pregnancies were consecutively enrolled in the present study. Women with multiple pregnancies (two or more fetuses), women who underwent fertility treatment, women with chronic diseases, gestational complications, systemic, and/or local infections that could prevent the participant from being physically active during pregnancy, women with a previous history of malignant, systemic, autoimmune, or musculoskeletal disorders, liver and renal disease, restrictive lung disease, women with psychiatric disorders, and those who did not speak Serbian fluently were excluded. Parity was defined as the number of children ever born to a woman and was categorized into three categories (0, 1, and ≥2). The study was conducted according to the guidelines of the World Medical Association's International Code of Medical Ethics (Declaration of Helsinki), and informed consent was obtained by mothers before participating in the study. This research was approved by the clinic's ethics committee (decision number: 05006-2021-1525; date of approval: September 17, 2020).

### 2.3. Data Collection

A general questionnaire was designed to collect sociodemographic and clinical information from participants. PA during pregnancy was assessed using the Serbian version of the Physical Activity Questionnaire during Pregnancy (PPAQ-SRB) [28], and depressive symptom severity was assessed using the Edinburgh Postnatal Depression Scale (EPDS) [29]. Both questionnaires have been linguistically adapted and standardized [29, 30].

The general questionnaire was related to sociodemographic characteristics (age, level of education, occupation, marital status, and socioeconomic status), clinical characteristics (general and reproductive health of the respondents, previous and current pregnancies), and lifestyle (smoking, alcohol consumption, and drug abuse).

The PPAQ-SRB was comprised of 33 questions in which respondents reported the time spent participating in various types of PA [28, 30]. A satisfactory level of reliability of the PPAQ-SRB was achieved (Cronbach's alpha coefficient = 0.69, and interclass correlation coefficient from 0.768 to 0.930) [28, 30]. All participants were interviewed by a qualified interviewer, and the answers were directly documented in the questionnaire.

The questionnaire included questions about household/caregiving, occupational and sports/exercise activities, transportation, and inactivity. The PPAQ questionnaire is an easy-to-use measurement tool as it assigns a value to each activity, expressed in metabolic equivalents (METS), per hour during the week. Participants reported the amount of time spent on each activity per day or week during the current trimester (response options ranged from 0 to 5, where 0 meant no PA at all, and 5 meant 3 or more hours a day). PA was calculated by multiplying the duration of each of the mentioned 30 PAs by the average weekly energy consumption (MET·hr/week), according to the instructions of the authors of the original version of the questionnaire [30]. METS are units used to assess metabolic activity (oxygen consumption) during PAs; 1 MET for the needs of metabolism at rest (basic life functions) is approximately 3.5 mL of oxygen/kg/min [31]. The PA of participants was classified into sedentary activity (<1.5 MET), low-intensity activity (1.5 – <3.0 MET), moderate-intensity activity (3.0–6.0 MET), and high-intensity activity (>6.0 MET) [30].

EPSD is a screening tool used worldwide for assessing symptoms of PPD [32, 33]. The EPSD consists of 10 questions that identify symptoms of depression in women during the postpartum period. The specified scale is a self-assessment scale that objectively assesses depressed mood and symptoms of depression but does not establish a diagnosis of depression, for which a clinical examination and evaluation by an expert are necessary. The EPSD was adapted to the population of pregnant women in the puerperium period in the study by Odalovic et al. [29], where the Cronbach's alpha was 0.84 and 0.83 in pregnant and postpartum women, respectively, indicating good internal consistency of the Serbian version of the EPSD.

The EPSD questionnaire was completed by a trained interviewer, who interviewed the respondents by phone for a period of 6–8 weeks after delivery. Each statement in the questionnaire was evaluated on a four-point scale with degrees from 0 to 3 and was related to the level of depression in the last 7 days. The total score of the scale was obtained by summing all answers to the questionnaire, and the answers ranged from 0 to 30, with a higher score indicating a greater intensity of depressive symptoms. Score values <8 indicated a low probability of experiencing symptoms of depression. Scores from 9 to 11 were considered intermediate probabilities for the existence of symptoms of depression. Values of 12–13 indicated a high probability of depression symptoms. A threshold value of 14 was considered to indicate a high level of symptomatic depression, that is, the probable existence of PPD. A positive score (provided answers 1, 2, or 3 are marked) when answering Question 10 indicates that one is at suicide risk [34].

### 2.4. Data Analysis

Descriptive statistical methods were used to characterize patients' sociodemographic and clinical data. Continuous variables are presented as averages (±standard deviation), and percentages are used for categorical variables. The Mann–Whitney *U* test was used to compare PPAQ-SRB scores among persons with and without symptoms of PPD because the data were not normally distributed. Univariate logistic regression analysis (ULRA) and multivariate logistic regression analysis (MLRA) were used to explore the relationship between PPAQ-SRB scores and PPD symptoms. An MLRA was performed to adjust for potential confounding factors. All *p*-values were based on two-tailed tests, and significance was set at *p*  < 0.05. All statistical analyses were performed using Statistical Package software (version 23.0; IBM Corp., SPSS Inc., NY, USA).

## 3. Results

### 3.1. Demographic Characteristics of the Analyzed Sample

Table 1 presents the participants' basic descriptive parameters. The mean age of the participants was 29.5 years (SD = 4.7).

The participants had an average body mass index of 24.99 ± 3.76 kg/m^2^. The average gestational weight gain was 10.28 ± 6.17 kg. More than half of the participants were in their first pregnancy (*n* = 58), and almost one-third (*n* = 34) had at least one pregnancy in the past. Urban residences were dominant in the study group, accounting for 74.3% of the total population. In addition, 92.4% of participants were employed. The majority of participants had a high school diploma or a higher level of education. Of the respondents, 74.29% were married, and 69.52% considered their financial situation to be satisfactory. More than one-third of the participants were smokers, 19.0% consumed excessive alcohol, and none of them used drugs during pregnancy.

### 3.2. Sociodemographic and Clinical Characteristics of the Subjects Included in the Study Depend on the Presence or Absence of PPD Symptoms

There were no statistically significant differences between sociodemographic and clinical characteristics depending on the presence or absence of symptoms of PPD (Table 2).

The observed prevalence of depressive symptoms among respondents participating in the study was 10.5%.

### 3.3. Examining the Significance of the Difference in PA through PPAQ-SRB Scores Depending on the Occurrence of PPD Symptoms

Women with symptoms of PPD had a significantly lower “Total score” of PPAQ-SRB (*p* < 0.001) and “Total activity (PA of light-intensity and above)” (*p* < 0.001). In addition, significantly lower scores were observed for PA of light-intensity (*p*=0.002), moderate-intensity (*p* < 0.001), “Household/care” (*p*=0.004), and “Occupational activities” (*p*=0.008) in women with symptoms of PPD (Table 3).

### 3.4. Univariate and Multivariate Logistic Regression between PPAQ-SRB Scores and the Occurrence of PPD Symptoms

ULRA results showed a significant association between the occurrence of PPD symptoms and the “Total score” of PPAQ-SRB (*p*=0.001) and “Total activities (PA of light-intensity and above)” (*p*=0.001). Also, a significant correlation was found between PA scores of light- (*p*=0.006) and moderate-intensity (*p*=0.003), as well as “Household/care” (*p*=0.015) and “Occupational” activities (*p*=0.017), and the occurrence of PPD symptoms. These associations remained significant after performing MLRA, which controlled for possible confounding factors (Table 4).

## 4. Discussion

In this prospective study following mothers during pregnancy and up to 8 weeks after delivery, we found lower levels of postnatal depression in more physically active women.

Our subjects who exhibited depressive symptoms spent fewer MET hours per week and were less physically active overall. Interestingly, our participants spent almost the same number of MET hours per week being inactive suggesting that mere inactivity is insufficient for PPD development. The lack of specific aspects of daily or weekly routines, as well as their level of intensity, can be indicative of PPD. Specifically, women with depressive symptoms in our study engaged in fewer activities that required low to moderate effort and were less active in both their households and work environments.

The results of our analysis showed that PA is a protective factor against the development of signs of depression in postpartum women. This is in accordance with previous reports indicating that PA during pregnancy, compared to inactivity, reduces the risk of developing PPD [27, 35, 36, 37]. Particularly, studies conducted in Europe demonstrated a significant correlation between PA and reduced PPD risk [27]. A cross-sectional Spanish study showed that pregnant women with no signs of depression were much more active [38]. A large French study by Van Der Waerden et al. [39] reported that high total PPAQ scores were inversely associated with the odds of depressive symptoms in the first postpartum year. Haßdenteufel et al. [40] showed that women who presented a greater reduction in PA levels during the course of pregnancy reported higher anxiety and depression symptom scores throughout the 6-month postnatal period, indicating that a stable level of total activity during pregnancy was significantly associated with a reduced probability of PPD occurrence. A Swedish study by Ekelöf et al. [41] reported that a higher level of PA during pregnancy was associated with a lower level of depressive symptoms postpartum. Therefore, PA is an essential element of a healthy lifestyle for women during childbirth [35].

Looking at PA intensity levels, our study showed that a decrease in light-to-moderate-intensity PA was crucial for the development of depressive disorders, as high-intensity levels were low overall in both groups. A study conducted in Spain revealed that women who performed moderate physical exercise in an aquatic environment during pregnancy were at lower risk of PPD than sedentary women [42]. Women who performed ≥150 min/week of moderate to vigorous PA had a significantly lower risk of PPD symptoms compared to those who did not accumulate any PA of at least moderate intensity [43]. Furthermore, as this study was conducted during the COVID-19 pandemic, unforeseen circumstances such as lockdowns and movement restrictions may have contributed to these results through the adoption of new habits when it comes to being intensely active. Therefore, the observed decline in light-to-moderate PA was significant, as moderate exercise is recognized as a potential protective factor associated with lower PPD symptom incidence during the pandemic [44]. Kołomańska-Bogucka et al. [45] compared the levels of light- and medium-intensity PA during late pregnancy before and after the onset of the pandemic. The study revealed a decrease in both categories during the pandemic, with particular emphasis on the impact of diminished light-intensity PA as a predictor for the onset of PPD symptoms, particularly during the pandemic. The authors reported that patients diagnosed at risk of developing depressive disorders had significantly reduced overall mobility and lower MET hours per week during the pandemic [45].

We did not observe significant differences in sedentary time between women with and without PPD, suggesting that passive leisure time is not a predictor of PPD onset. This is important because it contradicts the literature data, as it was previously reported that this type of sedentary behavior in the third trimester was associated with increased odds of PPD occurrence [39]. Furthermore, a systematic review revealed that higher sedentary behavior in the second trimester of pregnancy was likely to be associated with PPD and that longer sitting time may increase the risk of PPD symptoms [46].

Most women in our study engaged in light-to-moderate-intensity activities, either in their occupational or domestic settings, as most were employed. Other studies investigating domains of PA also yielded comparable outcomes, as the examined cohorts also spent most of their total PA in domestic settings [38, 45] or both home and work environments [39].

Our results indicated that higher levels of PA in occupational settings were associated with a lower incidence of PPD symptomatology. Similarly, another study demonstrated that when assessing PA using the PPAQ during the postpartum period, higher scores were associated with a reduced likelihood of developing PPD [39]. The obtained outcome was partly anticipated, as a literature review by Daley et al. [47] highlighted that occupational PA could be a useful strategy in lowering PPD occurrence. Occupational PA helps individuals focus on events other than their specific life circumstances, particularly daily hassles and stressful stimuli. Hence, the “occupational” PPAQ domain of our cohort was significant, as its low scores were a major predictor of screening positive for PPD. In contrast to our results, a dose–response meta-analysis of 186,412 women reported that work activities contributed to a greater risk of PPD [38]. Furthermore, our data suggested that higher levels of domestic and household PA were associated with lower EPDS scores. Recent investigations have also confirmed that household activities are the most energy-consuming PA domain during pregnancy [38, 48]. The available literature offers heterogeneous results when addressing high domestic activity as a risk factor for PPD development. Studies have shown that higher levels of household or caregiving in the third trimester were exclusively associated with increased odds of PPD [27, 39]. As lower scores in this domain were a risk factor for PPD in our cohort, the cultural aspects of PA habits were highlighted. Cultural differences related to the importance of childcare and homecare could impact new mothers' views that could be further reflected in this PPAQ domain, as there is generationally established knowledge that a well-established home environment and sufficient mother-care interactions have an impact on children's physical, cognitive, and social–emotional development [49]. Scores in the sports/exercise domain were not associated with PPD symptoms in this study. Studies that used the PPAQ to evaluate this domain of PA in postpartum women also found similar results, where a significant correlation between exercise alone during pregnancy and mental health during postpartum was not evident [39, 40, 45]. This contradicts the common notion, as a large meta-analysis showed that sports activities were associated with relieving symptoms [27]. When aerobic and strengthening exercises were tested on mothers who did not exercise before pregnancy and who initially had a positive score indicating the existence of depressive symptoms during pregnancy, they showed a major reduction in their EPDS score (below the score of 12, used as a threshold for depression) during the postpartum period compared with the control group that did not exercise [50]. Coll et al. [51] reported that low compliance with the exercise protocol, particularly among young and less-educated women, could be one of the main reasons why PA has not realized its full potential as a therapeutic and preventive intervention for PPD. It is possible that women reduced or discontinued their sports activities during late pregnancy, as it was shown that sports/exercise domains contributed relatively little to the total activity score, especially during the last trimester of pregnancy [39]. However, there is a need for PA domain exploration in further research, as a limited number of studies have examined the impact of antenatal PA domains on PPD.

Strengths of our study include prospective design, the use of reliable and valid assessment tools for evaluation of PA during pregnancy and symptoms of PPD, and consideration of a variety of important confounding factors such as age at delivery, place of residence, employment, socioeconomic conditions, BMI, and gestational weight gain. We determined the frequency, duration, and intensity of each PA domain and investigated their associations with PPD symptoms, which have been rarely studied in this population.

On the other hand, this study has limitations as well, including the use of self-report instruments to assess PA and symptoms of PPD, which may introduce some degree of misclassification. Although both questionnaires demonstrated good measurement properties, clinical assessment of PPD and PA measurements using an accelerometer or a heart rate monitor could provide more objective estimates. Furthermore, our findings may have been subject to residual confounding because we were unable to assess additional potential non-measured confounders, such as stress and sleep quality. Finally, our study was conducted using a small population from a single center with homogeneous characteristics, which may limit the generalizability of our results.

## 5. Conclusions

To our knowledge, this is one of the very few studies exploring the association between PA domains during pregnancy and PPD symptoms. The results of our study are consistent with previous studies showing that PA during pregnancy may improve depressive mood and symptoms during the postpartum period. In addition, lower levels of light-to-moderate-intensity household and occupational activities were associated with an increased risk of PPD, indicating that contextual factors may influence the way in which PA is correlated with mood and well-being. Our findings may contribute to the development of potentially viable strategies for preventing depression in new mothers, as well as the refinement of public health recommendations regarding appropriate PA engagement during pregnancy for postpartum mental health benefits.

## Figures and Tables

**Table 1:** Descriptive parameters of participants.

Parameter	*N* = 105 *x* ± SD
Maternal age at delivery (years) (range 19–40)	29.53 ± 4.73
BMI (range 17.11–37.96)	24.99 ± 3.76
Average gestational weight gain (kg) (range 2–25)	10.28 ± 6.17

	Numbers (%)

Residence:	
Urban	78 (74.3)
Rural	27 (25.7)
Employed:	
Yes	97 (92.4)
No	8 (7.6)
Education:	
Elementary school	1 (0.95)
High school	45 (42.85)
Undergraduate education	14 (13.33)
Faculty and postgraduate	45 (42.85)
Marital status:	
Married	78 (74.29)
Living with a partner but unmarried	24 (22.86)
Divorced	1 (0.95)
Single	2 (1,90)
Socioeconomic status:	
Poor	1 (0.95)
Average	31 (29.52)
Good	73 (69.52)
Parity:	
0	58 (55.24)
1	34 (32.38)
≥2	13 (12.38)
Lifestyle:	
Smoking during pregnancy	41 (39)
Alcohol during pregnancy	20 (19.0)
Drugs during pregnancy	0 (0)

SD, standard deviation; BMI, body mass index.

**Table 2:** Sociodemographic and clinical characteristics of women with and without PPD.

Variables	Without symptoms of PPD *N* = 94 *N* (%)	With symptoms of PPD *N* = 11 *N* (%)	*p*-Value
Maternal age at delivery			
19–25	20 (21.3)	3 (37.3)	0.594
26–30	33 (35.1)	4 (36.4)	
31–40	41 (43.6)	4 (36.4)	
BMI ≥ 25 (kg/m^2^)	46 (48.9)	3 (27.3)	0.185
Average gestation weight gain >10 kg	37 (39.4)	5 (45.5)	0.697
Urban residence	69 (73.4)	9 (81.8)	0.549
Employed	86 (91.5)	11 (100.0)	0.989
Higher education	53 (56.4)	6 (60.0)	0.826
Married	69 (73.4)	9 (81.8)	0.549
Good socioeconomic conditions	66 (70.2)	7 (63.6)	0.655
Smoking	35 (37.2)	6 (54.5)	0.272
Alcohol consumption during pregnancy	18 (19.1)	2 (18.2)	0.938
Nulliparity	51 (54.3)	7 (63.6)	0.556
Hospitalization during pregnancy	9 (9.6)	1 (9.1)	0.959

*p*-Value based on univariate logistic regression analysis; BMI, body mass index.

**Table 3:** Physical activity PPAQ-SRB scores for women with or without symptoms of PPD.

Variables unit: ^1^MET·hr/week	Without symptoms of PPD, *N* = 94	With symptoms of PPD, *N* = 11	*p*-Value
Total score of PPAQ-SRB^2^	42.31 ± 13.58	25.04 ± 6.41	**<0.001** *⁣* ^*∗∗*^
Total activity (light and above)	38.23 ± 13.71	20.19 ± 6.76	**<0.001** *⁣* ^*∗∗*^
By intensity			
Sedentary	4.08 ± 2.70	4.84 ± 2.92	0.393
Light	17.10 ± 7.02	10.47 ± 6.06	**0.002** *⁣* ^*∗*^
Moderate	20.84 ± 10.62	9.59 ± 5.28	**<0.001** *⁣* ^*∗∗*^
Vigorous	0.28 ± 0.42	0.12 ± 0.13	0.218
By type			
Household/caregiving	15.03 ± 10.88	5.41 ± 3.04	**0.004** *⁣* ^*∗*^
Occupational	16.07 ± 7.53	10.09 ± 4.49	**0.008** *⁣* ^*∗*^
Sports/exercise	0.98 ± 1.15	0.46 ± 0.57	0.102
Transportation	4.85 ± 2.96	3.34 ± 1.89	0.073
Inactivity	5.37 ± 3.74	5.73 ± 3.63	0.695

^1^МЕТ·hr/week, metabolic equivalent task · hours/week; ^2^PPAQ-SRB, Pregnancy Physical Activity Questionnaire-Serbian version; *p*-Value based on Mann–Whitney *U* test; *⁣*^*∗*^statistical significance *p* < 0.05; *⁣*^*∗∗*^statistical significance *p* < 0.001.

**Table 4:** Univariate and multivariate logistic regression between PPAQ-SRB scores and the occurrence of PPD symptoms.

Variables unit: ^1^MET·hr/week	Unadjusted OR (95% CI)	*p*-Value	Adjusted OR (95% CI)^†^	*p*-Value
Total score of PPAQ-SRB^2^	0.86 (0.78–0.94)	**0.001** *⁣* ^*∗*^	0.81 (0.70–0.93)	**0.03** *⁣* ^*∗*^
Total activity (light and above)	0.85 (0.77–0.94)	**0.001** *⁣* ^*∗*^	0.82 (0.71–0.93)	**0.03** *⁣* ^*∗*^
By intensity				
Sedentary	1.10 (0.88–1.39)	0.385	1.12 (0.85–1.47)	0.417
Light	0.83 (0.73–0.95)	**0.006** *⁣* ^*∗*^	0.81 (0.69–0.96)	**0.013** *⁣* ^*∗*^
Moderate	0.85 (0.76–0.84)	**0.003** *⁣* ^*∗*^	0.82 (0.71–0.94)	**0.005** *⁣* ^*∗*^
Vigorous	0.10 (0.00– 3.78)	0.213	0.04 (0.001–2.60)	0.133
By type				
Household/caregiving	0.85 (0.74–0.97)	**0.015** *⁣* ^*∗*^	0.85 (0.73–0.98)	**0.028** *⁣* ^*∗*^
Occupational	0.87 (0.78– 0.97)	**0.017** *⁣* ^*∗*^	0.80 (0.78–0.95)	**0.012** *⁣* ^*∗*^
Sports/exercise	0.44 (0.14–1.39)	0.164	0.37 (0.11–1.16)	0.089
Transportation	0.77 (0.55–1.07)	0.116	0.70 (0.46–1.04)	0.074
Inactivity	1.03 (0.87–1.21)	0.761	1.04 (0.85–1.27)	0.717

^†^Adjusted for age at delivery, place of residence, employment, socioeconomic conditions, BMI, gestational weight gain, ^1^MET·hr/week, metabolic equivalent task · hours/week; ^2^PPAQ-SRB, Pregnancy Physical Activity Questionnaire-Serbian version, OR, odds ratio; CI, 95% confidence interval; *⁣*^*∗*^statistical significance *p* < 0.05.

## Data Availability

The data that support the findings of this study are available from the corresponding author upon reasonable request.

## Abbreviations

PA: Physical activity
PPD: Postpartum depression
PPAQ-SRB: Pregnancy Physical Activity Questionnaire-Serbian version
EPDS: Edinburgh Postnatal Depression Scale
DSM-IV: The Diagnostic and Statistical Manual of Mental Disorders-Fourth Edition
ICS-10: International Classification of Diseases-Tenth Revision
RCT: Randomized controlled trial
MET: Metabolic equivalent task
SPSS: Statistical Package for Social Sciences
IBM: International Business Machines Corporation
SD: Standard deviation
BMI: Body mass index
ULRA: Univariate logistic regression analysis
MLRA: Multivariate logistic regression analysis
OR: Odds ratio
CI: Confidence interval
COVID-19: Coronavirus disease.
